# Cocaine Cue-Induced Dopamine Release in Recreational Cocaine Users

**DOI:** 10.1038/srep46665

**Published:** 2017-04-26

**Authors:** Sylvia M. L. Cox, Yvonne Yau, Kevin Larcher, France Durand, Theodore Kolivakis, J. Scott Delaney, Alain Dagher, Chawki Benkelfat, Marco Leyton

**Affiliations:** 1 Department of Psychiatry, McGill University, Montreal, Canada; 2 Department of Neurology and Neurosurgery, McGill University, Montreal, Canada; 3 Department of Emergency Medicine, McGill University, Montreal, Canada

## Abstract

It has been proposed that the acquisition of drug seeking is related to the development of conditioned dopamine responses in the ventral striatum. As drug use continues and becomes habit-like, conditioned responses have been shown to shift to the dorsal striatum. Here, using the PET [^11^C]raclopride method and highly personalized cocaine cues, we report the first evidence in humans of the dorsal dopamine response prior to the onset of addiction.

Neutral stimuli paired with cocaine can come to elicit striatal dopamine release. This response is thought to reflect the increased ability of cues associated with drug ingestion to promote incentive motivational states, including the reinstatement of drug-seeking behaviours^1^,^2^.

The exact locus of these striatal dopamine responses can change. Following relatively little cocaine use, the clearest drug cue-induced responses are seen in the ventral striatum^3^,^4^. Following more extensive cocaine use and the establishment of stimulus-response habits the largest responses are seen in the dorsal striatum^3^,^4^,^5^. An accumulation of these habits has been proposed to promote compulsive drug use and susceptibility to addiction^6^.

In humans, there is some evidence that these same transitions occur. Stimulant drug-related cues provoke dopamine release in the ventral striatum of healthy volunteers following exposure to only three prior doses of amphetamine^7^ and in the dorsal striatum of those with cocaine use disorders^8^,^9^,^10^. What remains unknown is whether dopamine responses can be seen in the dorsal striatum prior to the onset of an addiction. To investigate this, we used positron emission tomography (PET) with [^11^C]raclopride to measure dopamine responses to cocaine-paired cues in recreational cocaine users without a substance use disorder.

Cocaine users were interviewed in-depth with the structured clinical interview for DSM-IV-TR to rule out the presence of psychiatric disorders including addictions. Volunteers who met criteria for inclusion (lifetime cocaine use: 71 ± 60 occasions, see on-line methods and Table 1) underwent three test sessions on separate days. On session 1, participants engaged in a motor control task (typing a short paragraph for 3–5 minutes) and then, during a 60-min PET [^11^C]raclopride scan, watched a 60-min video of someone typing. On session 2, participants returned to the PET Unit with a friend that they used cocaine with. They were then videotaped while self-administering cocaine hydrochloride powder together (4 mg/kg/person, intra-nasal). From this footage, two 60-min videos were created, one per person. On session 3, subjects returned to the PET Unit and were presented with another bag of cocaine powder (4 mg/kg) that they could ingest immediately after the scan. After manipulating the powder into four lines, subjects were scanned while watching the video of their friend taking cocaine.

Exposure to the cocaine-related cues increased craving scores, as indicated by a significant cue (neutral or cocaine) x time interaction (F(3,24) = 4.0, p = 0.02, see Fig. 1). This effect was strongest immediately after manipulation of paraphernalia at the beginning of the video (t(8) = 2.27, p = 0.05) and at the end of the scan (t(8) = 2.4, p = 0.04), prior to the opportunity to self-administer cocaine. Exposure to the cocaine cues also decreased [^11^C]raclopride BP_ND_ values with statistically significant effects in the dorsal caudate (peak t = 6.56, MNI coordinates: −17, 12, 14, cluster size = 387 mm^3^) and dorsal putamen (peak t = 7.31, MNI coordinates: −31, −11, 9, cluster size = 994 mm^3^) but not in the ventral striatum (Fig. 2).

Together, these findings demonstrate that exposure to highly personalized cocaine related cues that lead to the opportunity to use the drug increases extracellular dopamine levels in the dorsal striatum in recreational cocaine users, providing the first evidence that this effect can be seen prior to the onset of a substance use disorder. An accumulation of dorsal striatum related habits, modulated further by motivational processes, is thought to increase susceptibility to compulsive drug use and addictions^1^,^2^,^6^,^11^.

Our conclusions need to be interpreted in light of the following considerations. First, the sample size was modest, which may have precluded seeing a smaller cue-induced dopamine response in the ventral striatum. Second, the PET [^11^C]raclopride method is limited to confidently measuring binding potential values within the striatum. It is considered likely, though, that drug-seeking behaviour, either goal-directed or habitual, is mediated by wider striato-nigro-striatal networks allowing information flow between functional cortical basal ganglia circuits^2^,^12^. Third, our sample included light smokers (five out of nine). Although they did not meet criteria for tobacco dependence, and all scored 0 out of 10 on the Fagerstrom Test for Nicotine Dependence, we cannot fully rule out a contribution to the cocaine cue-induced responses. These features noted, the present study provides the first demonstration that exposure to cocaine related cues increases extracellular dopamine levels in the dorsal striatum in recreational cocaine users. Dopamine transmission in this region might contribute to the development of persistent and excessive drug use.

## Methods

### Participants

Participants were recruited as pairs of cocaine-using friends selected from over 1000 interviewed volunteers responding to advertisements placed in local newspapers. All participants were male, free of current or past substance dependence, as determined by a semi-structured clinical interview for DSM-IV diagnoses^13^, and their primary and preferred route of cocaine use was intranasal (mean age 23.7 years, s.d. 6.1, range 18–38, see Table 1). Twelve volunteers (six pairs) who met criteria for inclusion were identified, eight for the neuroimaging study and four for a behavioural pilot study. Among these 12, one chose to be in session 2 only and two withdrew following session 2. Complete neuroimaging data were available from six participants, behavioural data were available from nine. One participant met criteria for past alcohol abuse and current cannabis abuse, and one met criteria for past cannabis abuse and past cocaine abuse (the latter completed the behavioural study only). Five of the nine participants were current light, social smokers with a score of 0 on the Fagerstrom Test for Nicotine Dependence^14^. Participants were free of other current Axis I psychopathology and were physically healthy as determined by a medical exam, electrocardiogram, and standard laboratory blood tests.

Before each test session, participants abstained from nicotine for at least 12 hours and from alcohol for at least 24 hours. On the morning of each test day, all tested negative on a urine drug screen sensitive to cocaine, opiates, phencyclidine, barbiturates, Δ^9^-tetrahydrocannabinol, benzodiazepines, and amphetamines (Triage Panel for Drugs of Abuse, Biosite Diagnostics, San Diego, California). The study was carried out in accordance with the Declaration of Helsinki and was approved by the Research Ethics Board of the Montreal Neurological Institute. All participants gave written informed consent.

### Procedure

The experimental procedure consisted of three test sessions. On session 1, participants engaged in a control task (typing a short paragraph for ~3–5 minutes), immediately followed by a positron emission tomography (PET) [^11^C]raclopride scan during which they viewed a 60-min control video (someone typing). On session 2, pairs of friends came back to the same PET environment where they were videotaped while ingesting cocaine hydrochloride together (4.0 mg/kg/person). Participants were instructed to prepare the dose into a minimum of four lines and then ingest it at their own pace. Two video cameras with audio were used, one per person. Each video was then edited into 60 minutes of material. On the third session, participants returned to the PET Unit alone. They were provided with cocaine powder (4.0 mg/kg), manipulated it into four or more equal lines, and, immediately afterward, underwent a PET [^11^C]raclopride scan during which they viewed the 60-minute videotape of their friend using cocaine. Following the scan, they had the option to ingest the provided cocaine. To keep the physical amount of powder constant, active drug was mixed with lactose to yield a total of 400 mg. The procedure for the behavioural pilot version was identical with the exception that subjects underwent two sham scans.

Self-report questionnaires were measured at four time points during sessions 1 and 3:

(1) Baseline, immediately after arrival at the laboratory.

(2) At the start of the PET scan, after tracer injection, after object manipulation and at the start of the video presentation.

(3) Halfway through the PET scan.

(4) At the end of the PET scan.

During the self-administration sessions (sessions 2 and 3) a project-dedicated nurse and emergency medicine physician were present onsite to monitor subject safety, and a psychiatrist was on call. Following each self-administration session subjects remained overnight for observation. Prior to being discharged, participants were evaluated by an emergency medicine physician. There were no adverse events.

### Image Acquisition and Processing

Subjects were scanned on a Siemens ECAT HR+ PET scanner (CTI/Siemens, Knoxville, Tennessee) with lead septa removed (63-slice coverage, with a maximum resolution of 4.2-mm full width at half maximum (FWHM) in the center of the field of view). Immediately after the transmission scan 8–10.5 mCi of [^11^C]raclopride was injected as a bolus into the antecubital vein. Emission data were collected over 60 min in 26 timeframes of progressively longer duration. PET images were reconstructed using a 6 mm FWHM Hanning filter and corrected for movement^15^.

For anatomical co-registration, high-resolution (1 mm) T1-weighted magnetic resonance images (MRI) were obtained for all subjects on a 1.5-T Siemens scanner, using gradient echo pulse sequence (repetition time = 9.7 msec, echo time = 4 msec, flip angle = 12°, field of view = 250 and matrix 256 × 256). Each MR image was first pre-processed with the CIVET pipeline (version 1.1.9) (wiki.bic.mni.mcgill.ca/ index.php/CIVET) developed at the Montreal Neurological Institute (MNI) for fully automated structural image analysis^16^. The native MR volumes were corrected for image intensity non-uniformity^17^, and linearly and non-linearly transformed into standardized MNI space using automated feature matching to the ICBM152 template^18^. The MR image in MNI space was classified into white matter, gray matter and CSF^17^, and was automatically segmented using a probabilistic atlas based approach^19^. The spatial rigid-body transformation between the summed PET volume and the native MR image was estimated with normalized mutual information, and was used to position each individual’s MRI into the native PET space.

Parametric images were generated by computing [^11^C]raclopride binding potential (BP_ND_) at each voxel using a simplified kinetic model that uses the cerebellum as a reference tissue devoid of dopamine D2/3 receptors to describe the kinetics of the free and specifically bound ligand^20^. BP_ND_ expresses the relationship between the estimated density of available dopamine receptors (B_avail_), the dissociation constant of its target dopamine receptor (K_D_), and the free fraction of non-specifically bound tracer in the brain (F_ND_)^21^.


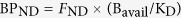


The changes in [^11^C]raclopride BP_ND_ between cocaine versus control videos were assessed at each voxel using a residual t-statistic^22^. A residual t-test improves the sensitivity to detect changes in BP_ND_ by incorporating an estimation of the standard deviation of the BP_ND_ map and by increasing the number of degrees of freedom (df = 2*N*(k − p), where N = number of subjects, k = number of frames, p = study parameters). Voxels of statistically significant change were identified by thresholding the *t* map at a value of *t* > 4.1, which corresponds to p < 0.05, Bonferroni corrected for multiple comparisons, a search volume of the entire striatum, df = 288 and a smoothing kernel of 6 mm at FWHM^23^.

### Self-Report Scales

Cocaine craving was assessed with a visual analogue scale (VAS) labeled “want cocaine” and ranging from 1 (least) to 10 (most).

### Statistics

All data were analyzed using repeated-measures analyses of variance (ANOVA), followed by planned comparisons as appropriate (SPSS version 22 software).

## Additional Information

**How to cite this article**: Cox, S. M. L. *et al*. Cocaine Cue-Induced Dopamine Release in Recreational Cocaine Users. *Sci. Rep.* **7**, 46665; doi: 10.1038/srep46665 (2017).

**Publisher's note:** Springer Nature remains neutral with regard to jurisdictional claims in published maps and institutional affiliations.

## Figures and Tables

**Figure 1:**
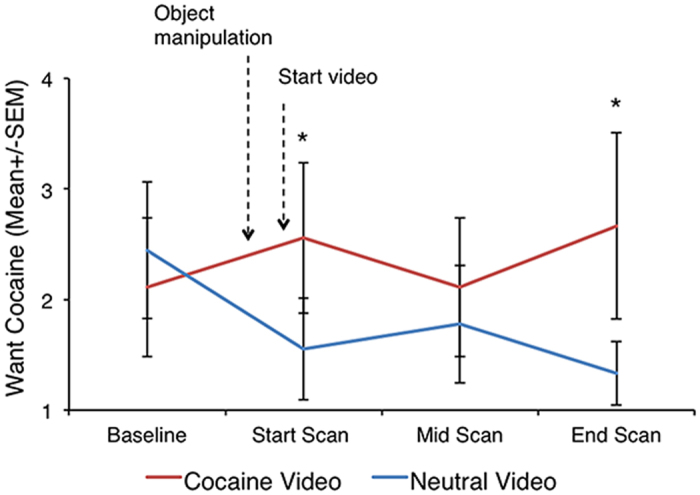
Cocaine-cue exposure increased self-reports of “Want Cocaine”. *Significantly different from neutral cue, p ≤ 0.05.

**Figure 2:**
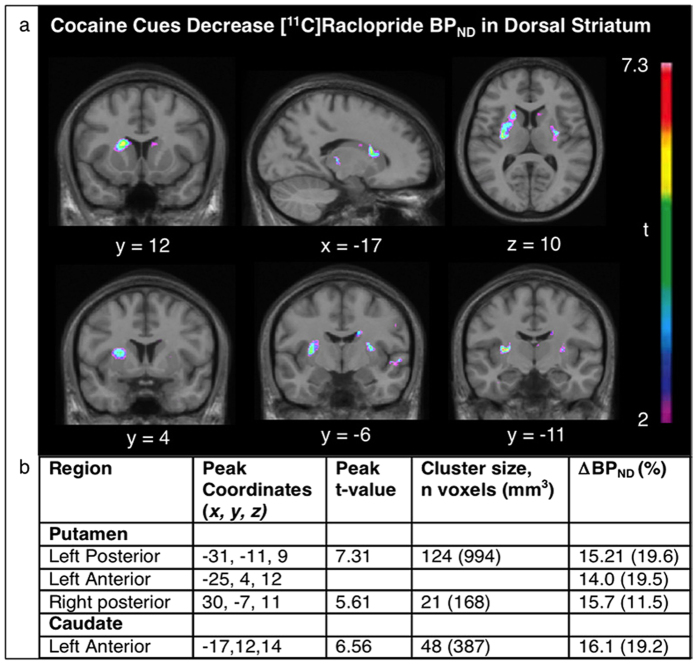
Cocaine cues decreased [^11^C]raclopride BP_ND_ in the dorsal striatum. (**a**) T-map illustrating significant decrease in [^11^C]raclopride BP_ND_ in response to cocaine *vs*. neutral cues (p < 0.05, corrected; significant at t > 4.1). (**b**) Regions showing significant decrease in [^11^C]raclopride BP_ND_ in response to cocaine *vs*. neutral cues (p < 0.05, corrected; significant at t > 4.1; cluster size n > 20 voxels).

**Table 1:** Drug use histories.

Substances Used	Occasions Used in Lifetime (n, mean ± SD)
Cocaine	
Occasions used, past 12 months	9/9, 28 ± 12.3 (range, 13–45)
Occasions used, lifetime	9/9, 71.4 ± 59.7 (range, 14–175)
Mean quantity used per occasion in past 12 months (gr)	9/9, 0.34gr ± 0.16gr (range, 0.25–0.7gr)
Amphetamine, occasions used, lifetime	4/9, 2.3 ± 1.7 (range, 0–5)
MDMA, occasions used, lifetime	8/9, 5.4 ± 3.8 (range, 0–12)
Opiates, occasions used, lifetime	0/9
Cannabis, occasions used, lifetime	9/9, 370 ± 373 (range, 12–1000)
Psilocybin, occasion used, lifetime	7/9, 9 ± 6.9 (range, 0–20)
LSD, occasions used, lifetime	3/9, 8 ± 10.4 (range, 0–20)
Ketamine, occasions used, lifetime	0/9
GHB, occasions used, lifetime	1/9, 2
Alcohol, occasions used, lifetime	9/9, 431 ± 250 (range, 70–850)*

^*^Missing lifetime use of alcohol data for 1 subject.
